# Supplementary material for: Inter-organizational alignment and implementation outcomes in integrated mental healthcare for children and adolescents: a cross-sectional observational study
Source: Implement Sci. 2024 May 27;19:36. doi: 10.1186/s13012-024-01364-w (PMC11129427; doi:10.1186/s13012-024-01364-w)
Supplement: Supplementary file 1 — Additional file 1. Embeddedness Ancillary Results. [file 13012_2024_1364_MOESM1_ESM.docx]

# Additional File 1: Ancillary Research Question 4 about Embeddedness

## Introduction: Clinicians’ Organizational Embeddedness

A basic premise underlying most integrated care (e.g., integrated SBMH) is that externally employed clinicians' organizational embeddedness (i.e., the extent to which clinicians are visible, connected to, and collaborate with local personnel in the service provision setting) can enhance youth's access to EBPs and subsequent outcomes [1, 2]. Also, clinician embeddedness is a reliable and amendable indicator of the extent and quality of clinicians' social and professional connections to local staff, leaders, and clients. In integrated SBMH, high levels of clinician embeddedness may enhance the impacts of the context factors in both CBOs (where training and supervision are conducted) and schools (where service is provided) on the outcomes of EBP implementation and clients. For instance, if a clinician is strongly embedded in the school as evidenced by healthy relationships with school personnel and a strong sense of belonging to the school, their implementation behaviors would be more responsive to and influenced by the levels of and alignment in the strategic implementation climate of their school and CBO [3,4]. Similarly, properly embedded clinicians interact more frequently with on-site leaders, so they would be more influenced about their leadership behaviors specific to EBP implementation. Thus, we hypothesized that the interplay between school and CBO-based context factors may be moderated by the levels of clinician embeddedness in integrated SBMH. Although severely underpowered, we followed the pre-registered study protocol to build 3-way interaction multilevel models (MLMs) to address an ancillary research question (RQ4) of "to what extent does *clinician embeddedness* moderate the *inter-dependent* *effects* between school- and CBO-based context factors on implementation outcomes?"

## Analysis

The 13-item subscale of *Outreach and Approach* *by Mental Health Professionals* was adopted from the Expanded School Mental Health Collaboration Instrument (ESCI) to assess the level of the CBO-employed clinicians' embeddedness in schools. For RQ4, we hypothesized that clinician embeddedness would further moderate the 2-way interactions between the CBO and school context factors from RQ 3. Hence, we examined the 3-way interaction effect among clinician embeddedness, CBO, and school context factors on implementation outcomes. Per the hierarchy principle, all possible pairs of 2-way interaction terms were entered to ensure the interpretability of the 3-way interaction term [5]. To facilitate readers to interpret the 3-way interaction effects, we selectively plotted two exemplary types of 3-way interactions (positive and negative; Figures 4 and 5).

## Results of 3-way Interaction Effects MLM

Like the 2-way interactions in RQ 3, for most implementation outcomes, the moderation effects of *Clinician Embeddedness* on the 2-way interaction between CBO and school strategic factors (i.e., 3-way interaction effects) were smaller than those of general factors. Among strategic context factors, the 3-way interaction effect for *Strategic Climate* was larger than that of *Strategic Leadership* on most implementation outcomes. Among general context factors, 3-way interaction effects for *Functionality* were the largest on most implementation outcomes. On the other hand, the 3-way interaction effects showed inconsistent directions across different types of context factors and implementation outcomes. In the case of different context factors for the same implementation outcome (e.g., *Treatment Integrity)*, *Clinician Embeddedness* boosted the 2-way interaction effect of *Strategic leadership* (Figure 1) but suppressed the effect of *Stress* (Figure 2). In the case of the same context factors for different implementation outcomes, *Clinician Embeddedness* bolstered the 2-way interaction effect between CBO and school *Strategic climate* on *Implementation Citizenship Behavior* (Figure 3), but it suppressed the 2-way interaction effect of *Strategic climate* on *Treatment Integrity* (Figure 4).

## Discussion: Clinicians' Organizational Embeddedness in Integrated Care

This study revealed mixed findings about how *Clinician Embeddedness* enhances or dampens the inter-dependent effects between CBO and school implementation context factors on implementation outcomes. In terms of effect sizes, we found that *Clinician Embeddedness* exerted a larger moderation effect on the interdependence (IOA) between CBO and school general factors than strategic ones. However, none of these moderation effects reached statistical significance, possibly due to our small sample and the power-consuming configuration of moderation MLMs. In most integrated SBMH settings in the US, considerable variation existed in the extent to which SBMH clinicians communicate, collaborate, and connect to personnel in schools where they are embedded [6]. Our null finding calls for future replication studies with a larger sample and adequate power to verify whether the 3-way interaction is truly non-significant among embeddedness and IOA between CBO and school context factors. If large-scale replication studies corroborated our finding, it would support the robust effect of the IOA between CBO and school context factors against varied *Clinician Embeddedness* on the implementation outcomes of integrated SBMH. Also, it could inform field practitioners and leaders who may want to strategically improve their IOAs in strategic factors as a protective factor against varied *Clinician Embeddedness* in their existing integrated SBMH.

Moreover, we found some negative 3-way interaction effects of *Clinician Embeddedness* on certain context factors and implementation outcomes. The seemingly counterintuitive finding might be interpreted in conjunction with the types of interdependent effects (i.e., 2-way interactions) between CBO and school context factors. Most of the negative moderation effects of *Clinician Embeddedness* were observed in models where the interdependent effects were also negative (i.e., suppressive effect). This means that higher levels of *Clinician Embeddedness "*keep in check" the suppressive effect of an implementation context factor in one setting on its effect in the other setting (see previous section). These findings shed light on the intricate and multi-dimensional interplay between clinician embeddedness and implementation contexts in integrated mental healthcare for children and adolescents, which warrants future research with a larger sample of organizations involved in integrated care.

# Reference

1. Woodard GS, Triplett NS, Martin P, Meza RD, Lyon AR, Berliner L, Dorsey S. Implementing mental health services for children and adolescents: caregiver involvement in school-based care. Psychiatric Services. 2020 Jan 1;71(1):79-82.
2. Lyon AR, Whitaker K, Locke J, Cook CR, King KM, Duong M, Davis C, Weist MD, Ehrhart MG, Aarons GA. The impact of inter-organizational alignment (IOA) on implementation outcomes: evaluating unique and shared organizational influences in education sector mental health. Implementation Science. 2018 Dec;13:1-1.
3. Palinkas LA, Fuentes D, Finno M, Garcia AR, Holloway IW, Chamberlain P. Inter-organizational collaboration in the implementation of evidence-based practices among public agencies serving abused and neglected youth. Adm Policy Ment Health Ment Health Serv Res. 2014;41:74–85.
4. Mellin EA, Weist MD. Exploring school mental health collaboration in an urban community: a social capital perspective. Sch Ment Heal. 2011;3:81–92.
5. Dawson JF, Richter AW. Probing three-way interactions in moderated multiple regression: development and application of a slope difference test. Journal of applied psychology. 2006 Jul;91(4):917.
6. Mellin EA, Taylor L, Weist MD. The expanded school mental health collaboration instrument [school version]: development and initial psychometrics. Sch Ment Heal. 2014;6:151–62.

# Figures


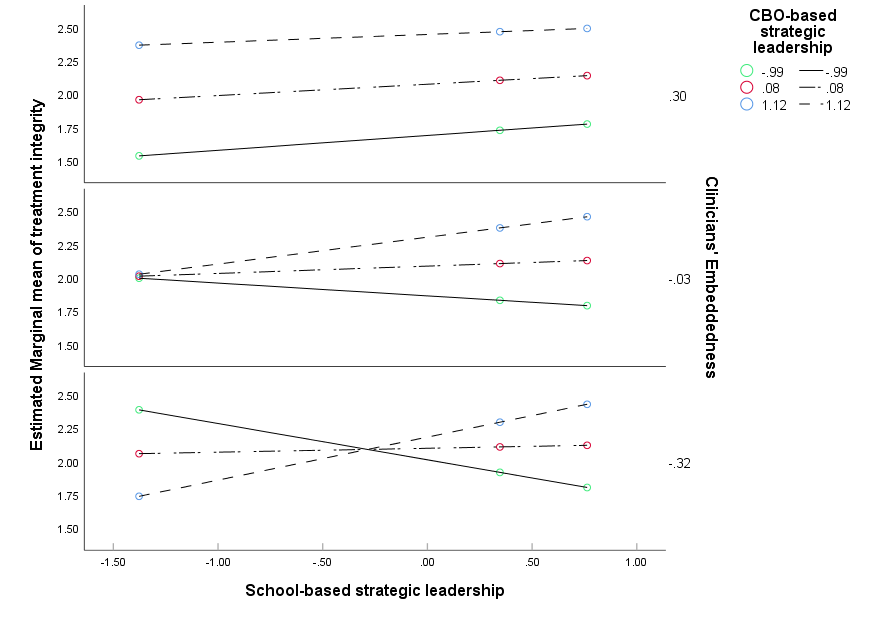


*Figure 1.* Example of the positive/compensatory 3-way interaction effect of Clinicians' Embeddedness on the 2-way interaction effect between CBO versus school context factors (Strategic leadership) on implementation outcomes (treatment integrity) in integrated mental healthcare. The predictors were group mean centered. The three plot panels laid out vertically= three levels of the 3-way moderator (Clinicians' Embeddedness; from up to bottom, 84^th^, 50^th^, and 16^th^ percentiles). Within each plot panel, black lines = smoothed regression lines for the three levels of the 2-way moderator (CBO-based Strategic leadership). Solid line with green dots = high level of moderator (84th percentile), long-dash line with red dots= moderate level of moderator (50th percentile), short-dash lines with blue dots= low level of moderator (16th percentile).


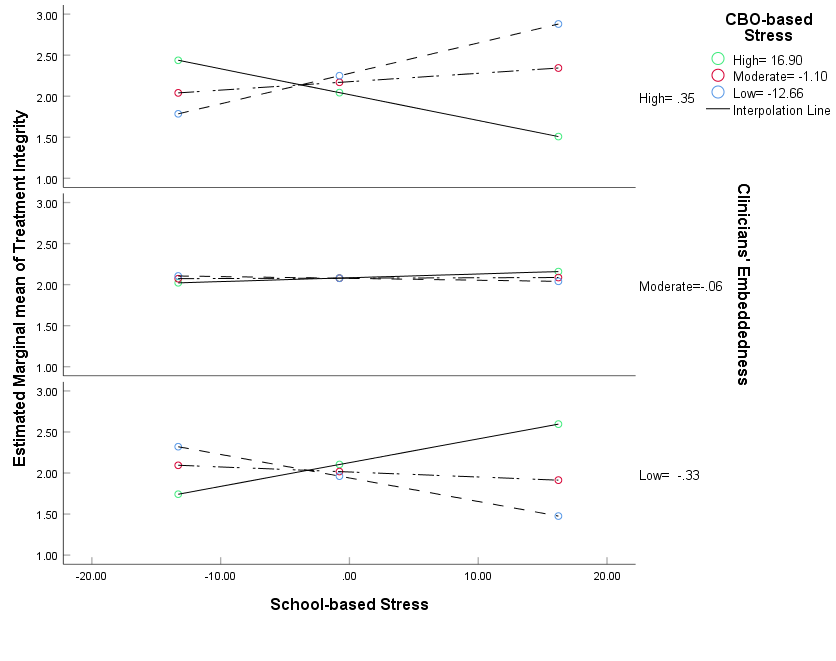


*Figure 2.* Example of the negative/suppressive 3-way interaction effect of Clinicians' Embeddedness on the 2-way interaction effect between CBO versus school context factors (general factor of Stress) on implementation outcomes (treatment integrity) in integrated mental healthcare. The predictors were group mean centered. The three plot panels laid-out vertically= three levels of the 3-way moderator (Clinicians' Embeddedness; from up to bottom, 84^th^, 50^th^, and 16^th^ percentiles). Within each plot panel, black lines = smoothed regression lines for the three levels of the 2-way moderator (CBO-based Stress). Solid line with green dots = high level of moderator (84th percentile), long-dash line with red dots= moderate level of moderator (50th percentile), short-dash lines with blue dots= low level of moderator (16th percentile).


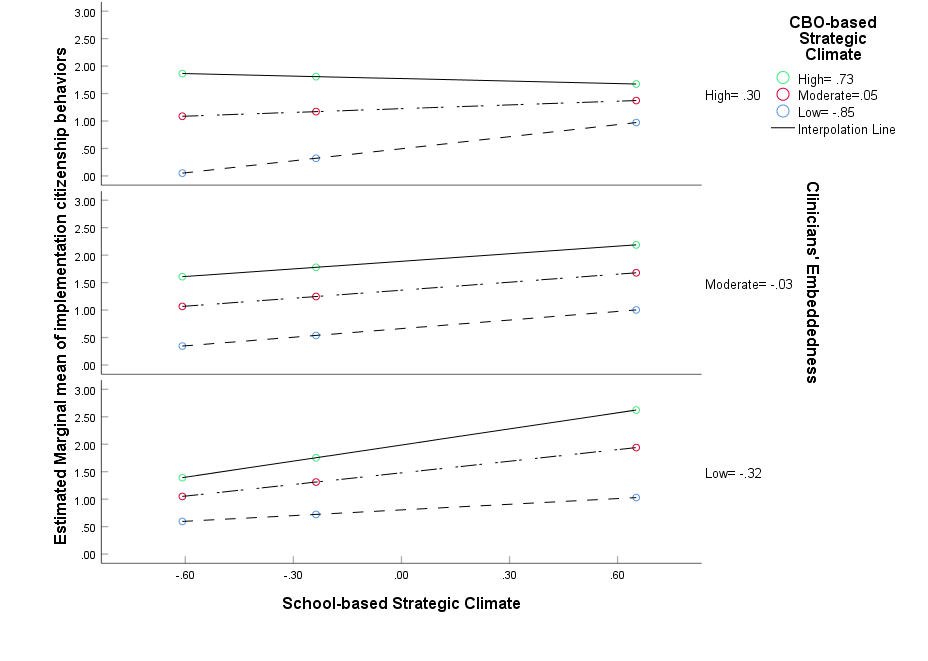


*Figure 3.* Example of the positive/compensatory 3-way interaction effect of Clinicians' Embeddedness on the 2-way interaction effect between CBO versus school context factors (Strategic Climate) on implementation outcomes (Implementation citizenship behaviors) in integrated mental healthcare. The predictors were group mean centered. The three plot panels laid-out vertically= three levels of the 3-way moderator (Clinicians' Embeddedness; from up to bottom, 84^th^, 50^th^, and 16^th^ percentiles). Within each plot panel, black lines = smoothed regression lines for the three levels of the 2-way moderator (CBO-based Strategic Climate). Solid line with green dots = high level of moderator (84th percentile), long-dash line with red dots= moderate level of moderator (50th percentile), short-dash lines with blue dots= low level of moderator (16th percentile).


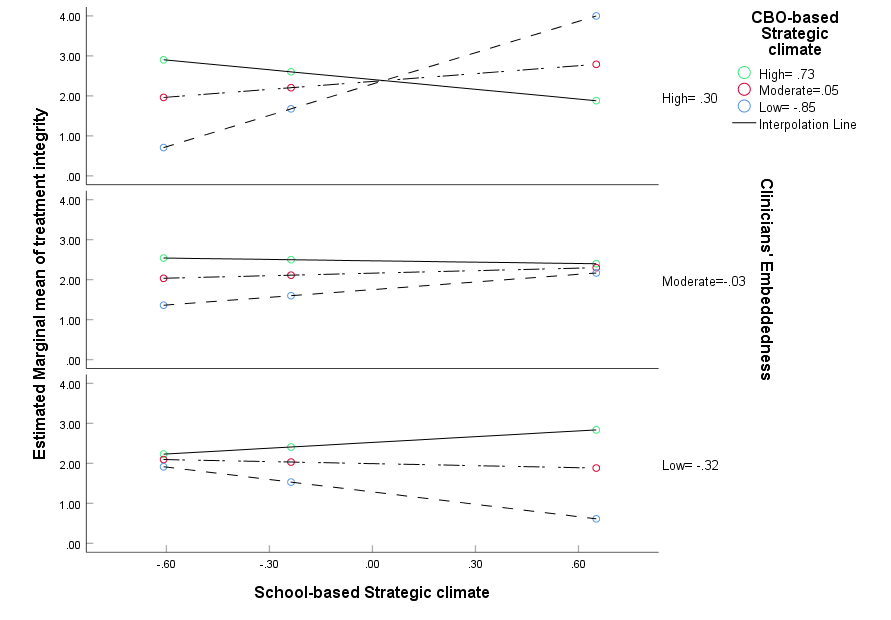


*Figure 4.* Example of the negative/suppressive 3-way interaction effect of Clinicians' Embeddedness on the 2-way interaction effect between CBO versus school context factors (Strategic Climate) on implementation outcomes (treatment integrity) in integrated mental healthcare. The predictors were group mean centered. The three plot panels laid-out vertically= three levels of the 3-way moderator (Clinicians' Embeddedness; from up to bottom, 84^th^, 50^th^, and 16^th^ percentiles). Within each plot panel, black lines = smoothed regression lines for the three levels of the 2-way moderator (CBO-based Strategic Climate). Solid line with green dots = high level of moderator (84th percentile), long-dash line with red dots= moderate level of moderator (50th percentile), short-dash lines with blue dots= low level of moderator (16th percentile).
