# Supplementary material for: Inter-organizational alignment and implementation outcomes in integrated mental healthcare for children and adolescents: a cross-sectional observational study
Source: Implement Sci. 2024 May 27;19:36. doi: 10.1186/s13012-024-01364-w (PMC11129427; doi:10.1186/s13012-024-01364-w)
Supplement: Supplementary file 2 — Additional file 2. Multilevel Model Equations. [file 13012_2024_1364_MOESM2_ESM.docx]

# Additional File 2: MLM Equations

**Variables In All Models:** There are **9** *Implementation Outcomes*, including *Treatment Integrity, Support for Teachers and Students, Increased Mental Health Programming, Improved Access for Students and Families,* *Acceptability, Appropriateness,* and *Feasibility, Implementation Citizenship Behavior,* and *Attitudes Toward EBPs.* There are **5** *Explanatory Variables*, including *Strategic Leadership, Strategic Climate*, Three indicators of *Organizational Social Context* (*Proficiency, Stress*, and *Functionality*). For RQ4, there is **1** moderator: *Clinician Embeddedness.* For subscripts in all equations, i= clinician/school ID, j= CBO ID.

The implementation outcome of *Treatment Integrity* contains four items that capture individual dimensions of treatment integrity. In addition to using the aggregated score for modeling (as planned in the study protocol), we explored the individual dimensions of treatment integrity by building GLMM using the individual items (ordinal scale data). However, the results were largely consistent with the model using total score that represents a holistic indicator of the multidimensional construct of treatment integrity. Hence, we did not report the extra models considering the clarity and conciseness of the study, as well as the limited meaningfulness of these ancillary results for our specific research question and future research.

**RQ1: Levels of Inter-Organizational Alignments**

**For ICC Calculations**

**Level 1 Equation**

*Implementation Outcome* *_ij_* = $\beta_{0}$+ $r_{ij}$

**Level 2 Equation**

$\beta_{0}$ = $\gamma_{00}$ $+$ $\mu_{0j}$

*Note.* For Inter-OA, 2-way mixed ICCs were calculated where the dyads of CBO- and school-based ratings of a context factor (level-1 units) were nested within clinicians (level-2 units).

### For Covariate Screening

**Level 1 Equation (*n* = *27*)**

*Implementation Outcome* *_ij_* = ${\beta_{0}+\beta}_{1}*\left( \mathrm{Race} \right)+\beta_{2}*\left( \mathrm{Age} \right)+\beta_{3}*\left( \mathrm{Gender} \right)+\beta_{4}*\left( Work Experience \right)$ + $\beta_{5}*\left( Educational level \right)$ + $r_{ij}$

**Level 2 Equation (*n = 9*)**

$\beta_{0}$ = $\gamma_{00}$ +$\mu_{0j}$

$\beta_{1}$ = $\gamma_{10}$

$\beta_{2}$ = $\gamma_{20}$

$\beta_{3}$ = $\gamma_{30}$

$\beta_{4}$ = $\gamma_{40}$

$\beta_{5}$ = $\gamma_{50}$

**RQ 2: Standalone Main Effect of Implementation Context Factors**

**Level 1 Equation (*n* = *27*)**

*Implementation Outcome* *_ij_* = ${\beta_{0}+\beta}_{1}*\left( CBO-based Explanatory Variable \right)+\beta_{2}*\left( School-based Explanatory Variable \right)+r_{\mathrm{ij}}$

**Level 2 Equation (*n = 9)***

$\beta_{0}$ = $\gamma_{00}$ $+$ $\mu_{0j}$

$\beta_{1}$ = $\gamma_{10}$

$\beta_{2}$ = $\gamma_{20}$

**RQ 3: 2-way Interaction between CBO- and School-Based Implementation Context Factors**

**Level 1 Equation (*n* = *27*)**

*Implementation Outcome* *_ij_* = ${\beta_{0}+\beta}_{1}*\left( CBO-based Explanatory Variable \right)+\beta_{2}*\left( School-based Explanatory Variable \right)+\beta_{3}*\left( CBO-\mathbf{X} School-based Explanatory Variables \right)+r_{\mathrm{ij}}$

**Level 2 Equation (*n = 9)***

$\beta_{0}$ = $\gamma_{00}$ + $\mu_{0j}$

$\beta_{1}$ = $\gamma_{10}$

$\beta_{2}$ = $\gamma_{20}$

$\beta_{3}$ = $\gamma_{30}$

**RQ 4: 3-way Interaction Among Clinician Embeddedness, CBO- and School-Based Implementation Context Factors**

**Level 1 Equation (*n* = *27*)**

*Implementation Outcome_ij_* = ${\beta_{0}+\beta}_{1}*\left( Clinician Embeddedness \right)+\beta_{2}*\left( CBO-based Explanatory Variable \right)+\beta_{3}*\left( School-based Explanatory Variable \right)+\beta_{4}*\left( CBO-\mathbf{X} School-based Explanatory Variables \right)+\beta_{5}*\left( Clinician Embeddedness \mathbf{X} CBO-based Explanatory Variables \right)+\beta_{6}*\left( Clinician Embeddedness \mathbf{X} School-based Explanatory Variables \right)+\beta_{7}*\left( Clinician Embeddedness \mathbf{X} CBO-\mathbf{X} School-based Explanatory Variables \right)+r_{\mathrm{ij}}$

**Level 2 Equation (*n = 9)***

$\beta_{0}$ = $\gamma_{00}$ + $\mu_{0j}$

$\beta_{1}$ = $\gamma_{10}$

$\beta_{2}$ = $\gamma_{20}$

$\beta_{3}$ = $\gamma_{30}$

$\beta_{4}$ = $\gamma_{40}$

$\beta_{5}$ = $\gamma_{50}$

$\beta_{6}$ = $\gamma_{60}$

$\beta_{7}$ = $\gamma_{70}$
